# Gene Variant Frequencies of *IDO1*, *IDO2*, *TDO*, and *KMO* in Substance Use Disorder Cohorts

**DOI:** 10.3390/genes15111388

**Published:** 2024-10-29

**Authors:** Lindsey Contella, Christopher L. Farrell, Luigi Boccuto, Alain Litwin, Marion L. Snyder

**Affiliations:** 1 Healthcare Genetics and Genomics, School of Nursing, Clemson University, 605 Grove Rd., Greenville, SC 29605, USA; 2 Luxor Scientific, LLC, 1327 Miller Rd., Greenville, SC 29607, USA; 3 School of Health Research, Clemson University, Clemson, SC 29631, USA; 4 Department of Medicine, Prisma Health, 701 Grove Rd., Greenville, SC 29605, USA; 5 Department of Medicine, University of South Carolina School of Medicine, 876 W Faris Rd., Greenville, SC 29605, USA

**Keywords:** kynurenine pathway, substance use disorder, tryptophan, genetic frequency

## Abstract

Background: Substance use disorder in the United States represents a complex and growing public health crisis, marked by increasing rates of overdose deaths and the misuse of prescription medications. There is a critical need for furthering the understanding of the molecular and genetic mechanisms that can lead to substance use disorder. Identifying significant variants in the kynurenine pathway could help identify therapeutic targets for intervention. Methods: The All of Us cohort builder evaluated the frequency of variants of four genes, *TDO2*, *IDO1*, *IDO2*, and *KMO*, encoding enzymes in the kynurenine pathway. The samples were broken into six cohorts: alcohol, cannabis, cocaine, opioid, other use disorder, and control. Using Chi-square analysis, the frequency of at least one copy of a variant allele was calculated. Results: Chi-square analysis showed a significant variation in genetic frequency (*p*-value < 0.005) in 14 of 18 polymorphisms analyzed. The cocaine cohort had the most significant variants (13), cannabis had 11, opioids had 3, other use disorders had 2, and alcohol had 1 significant variant. Conclusions: This study found associations of polymorphisms in the *TDO2*, *IDO1*, *IDO2*, and *KMO* genes of individuals with a substance use disorder. These results provide evidence of potential predictors of increased susceptibility to substance use disorder.

## 1. Introduction

Substance use disorder (SUD) currently presents as a significant public healthcare challenge impacting the United States population [1]. According to the National Survey on Drug Use and Health (NSDUH) conducted by the Substance Abuse and Mental Health Services Administration (SAMHSA) in 2019, approximately 20.4 million adults had a problem with addictive substances, which is continually growing in number [2]. SUD is a complex, multifaceted condition with genetic and environmental components [3]. Given the complexity of the disorder, current therapeutics are suboptimal, and more research is needed to advance precision medicine as it relates to the treatment of SUD.

Traditionally, the dopamine reward system has been a focus of research in SUD because of the known correlation between the release of dopamine and the intake of addictive drugs [2]. The addictive substance promotes the release of dopamine, causing the progression from drug use to drug-seeking behavior [4]. The dopamine reward system is closely linked to tryptophan (TRP) metabolism, as the metabolites of the breakdown of TRP can activate dopamine neurons. The two sister pathways that break down TRP are the serotonin pathway (SP) and the kynurenine pathway (KP) [5]. Both pathways have been identified as having a role in SUD. This research focuses on KP for three reasons: first, its role in neuroinflammation and neurotoxicity; second, the known correlation between KP and mood disorders, which are typical a comorbidity of SUD; and lastly, research is currently limited since it breaks down the majority of TRP, for which a better understanding of the pathway is needed [5,6,7]. Genetic variants can have multiple roles. They can influence metabolic pathways during substance use and also affect the neurobiology of substance dependence, which is mediated by complex neuronal circuits. These circuits, including dopamine, regulate reward, motivation, and reinforcement processes, and are critical in driving craving, desire, and impulsivity associated with substance use disorders [8].

**Figure 1:**
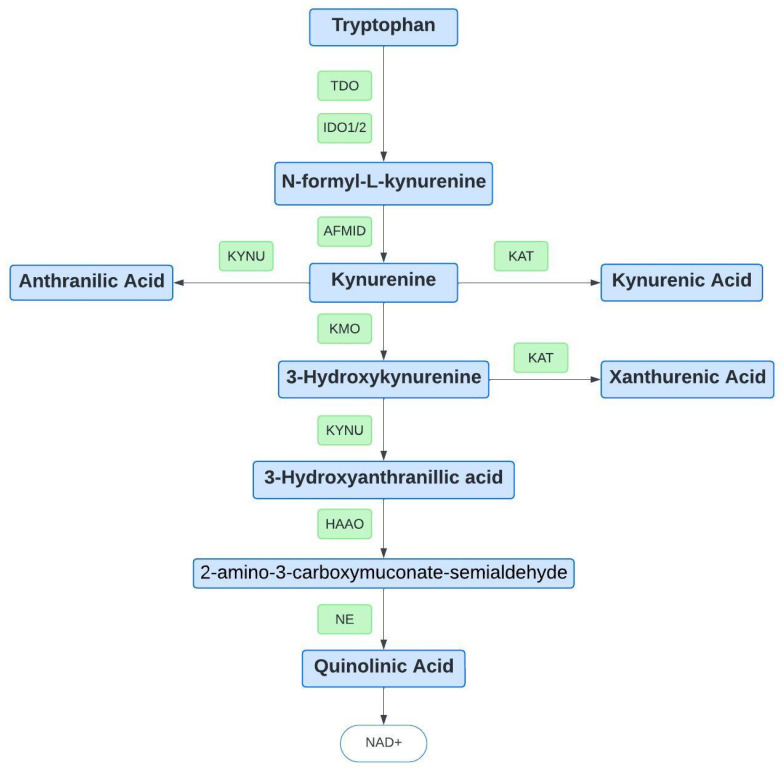
Tryptophan metabolism through the kynurenine pathway. Abbreviations: IDO1: indoleamine 2,3-dioxygenase-1, IDO2: indoleamine 2,3-dioxygenase-2, TDO: tryptophan 2,3-dioxygenase, NAD+: nicotinamide adenine dinucleotide.

The metabolism of TRP through the KP, where 95% of TRP is metabolized, is shown in Figure 1 [6]. The KP’s initial step involves converting TRP to N-formyl-L-kynurenine by the enzymes tryptophan 2,3-dioxygenase (TDO2) or indoleamine 2,3-dioxygenase (IDO1/2). This is a rate-limiting step of the process, controlling TRP and KP metabolite concentrations [9]. Subsequently, arylformamidase (AFMID) mediates the N-formyl-L-kynurenine conversion to kynurenine (KYN), which three different pathways can metabolize. The neuroprotective pathway uses kynurenine aminotransferases (KATs) to convert KYN to kynurenic acid (KYNA), or kynureninase (KYNU) converts KYN to anthranilic acid (AA), or kynurenine 3-monooxygenase converts KYN to 3-hydroxykynurenine (3-HK). KYNU metabolizes 3-HK to 3-hydroxy anthranilic acid (3-HAA) or by KATs to xanthurenic acid (XA). Next, 3-HAA can be converted into quinolinic acid (QA), a neurotoxic metabolite, in two steps, initially by 2-amino-3-carboxymuconate-semialdehyde by 3-hydroxy anthranilate 3,4-dioxygenase (HAAO) and subsequently by a non-enzymatic reaction. Lastly, QA can be further metabolized to synthesize nicotinamide-adenine-dinucleotide (NAD+), a molecule involved in energy storage and transfer within the cell. Each step plays a critical role in the process, and imbalances of these metabolites are a root cause for associated disease states. For example, having a balanced ratio of KYN and QA can impact depression, and recently, restoring the balance has been studied in preventing substance abuse relapse [10]. Identifying variants in the genes encoding the enzymes responsible for metabolite conversion can help identify genetic components to imbalances in the metabolites of the KP, leading to more precise therapeutic options.

Genetic variants of the KP have been studied in multiple disease states, including Crohn’s disease, Tourette syndrome, autism, Parkinson’s disease, depressive symptoms, substance use, and others [11]. Previously, Pisanu et al. identified a higher rate of variants in the *TDO*, *IDO1/2*, *KMO*, and *KAT* genes of patients diagnosed with bipolar disorder compared to a control group [12]. Multiple other studies found similar findings with a higher percent of variants in bipolar disorder, schizophrenia, and depressive disorder compared to control groups [13,14,15]. A comorbidity of SUD is mood disorders, suggesting the potential of a higher frequency of genetic variants in the KP in SUD as well [7]. Minimal research has been completed to identify alterations of the KP and its connection with SUD, but the limited data had conflicting results. Comings et al. found an association between SUD and polymorphisms in the gene that codes for the enzyme TDO2 [8]. Alternatively, Soichot et al. found no significant difference in variants in the promoter region of the *TDO2* gene in the alcohol use cohort compared to a healthy control cohort [9]. This finding highlights the need for further research to identify if there is an association between genetic variants of the enzymes of the KP.

In this study, we hypothesized that the frequency of genetic polymorphisms of three primary genes in the KP is higher in individuals diagnosed with SUD than in the general population. The primary aim of this work was to use the AoU data to determine the frequency of genetic polymorphisms in the enzymes of the KP, specifically *TDO2*, *IDO1/2*, and *KMO* genes. This study aims to confirm the existence of a correlation between KP gene variants and substance use disorder. A comprehensive understanding of the molecular genetics of substance use is essential for a greater understanding of the mechanisms that could provide further knowledge on therapeutics.

## 2. Methods

### 2.1. All of Us Database

The All of Us Research Program (AoU) is a source of genetic data that aims to gather health data on over a million people. The data include electronic health records (EHRs), laboratory results, physical measurements, survey responses, and genomic data. The data are a prospective cohort study initiated in 2018, aiming to enhance population-based research and deepen comprehension of human diseases, ideally improving therapeutic and treatment options to support precision medicine. As of March 2024, AoU has released whole-genome sequencing data for 245,400 participants. Informed consent was obtained from each participant enrolled in the AoU program. This dataset provides valuable information that can help identify correlations between genetic variants and disease states. The AoU data offer a database to research genetic variants in multiple disease states, including historically underrepresented populations and large sample sizes.

### 2.2. AoU Participant Selection

This study was performed on cohorts and genomic data from AoU participant data collected as previously described [16]. The AoU Controlled Tier Researcher Workbench was utilized to create cohorts for analysis. The AoU Researcher Workbench is a cloud-based platform where authorized researchers can access data from the AoU program. The Researcher Workbench allows for data analysis in Jupyter Notebooks using Python and R programming languages for statistical analysis. All data extracted in this process are stored within the workbench and are only accessible to researchers with approved researcher status. Institutional Review Board (IRB) was obtained at Clemson University (IRB 2024-0472).

**Figure 2:**
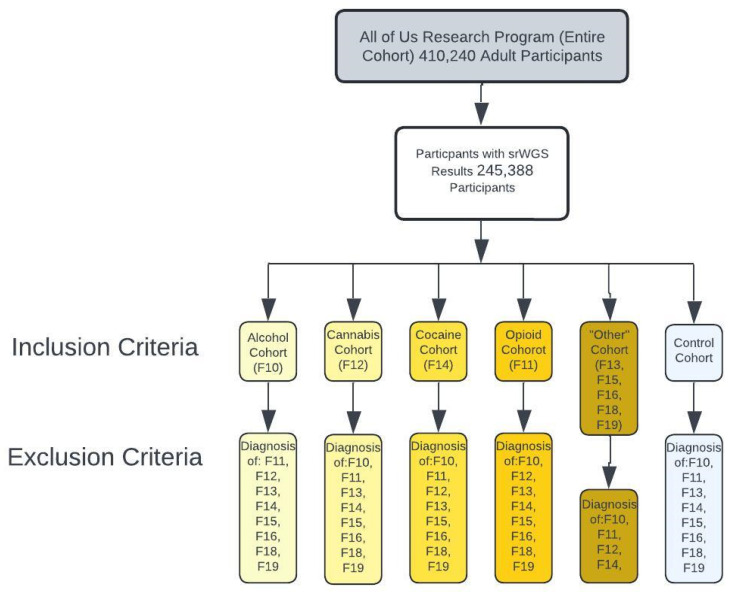
Study workflow and cohort definition for evaluating the frequency of genetic variants of the TDO2, IDO1/2 and KMO in substance use disorder.

The initial criteria to filter the entire AoU cohort were adults (>18 years of age at the moment of data collection) and participants with short-read whole-genome sequencing (srWGS) (*n* = 245,388). Next, this group of participants was broken into subgroups based on the International Classification of Disease (ICD) 10th revision codes. The ICD10 codes and their description are listed in Table 1. Cohorts were split into four groups: alcohol use disorder (F10) (*n* = 4832), cannabis use disorder (F12), cocaine use disorder (F14), opioid use disorder (F11) and other (F13, F15, F14, F18, and F19). The control cohort excluded participants from the previous cohorts with a diagnosis code associated with SUD. Inclusion and exclusion criteria are shown in Figure 2. This criterion was used to build cohorts. Dataset Builder was utilized to create participant datasets categorized by ICD-10 codes to extract each cohort’s demographic information and genomic frequency.

Demographic information was exported to a Python 3.0 notebook within the AoU workbench environment. During the export process, the AoU Workbench creates Python code and imports it directly into the notebook. Python was also used to summarize demographic information.

### 2.3. SNP Selection and Genotyping

When participants consented to participate in the AoU research program, a whole blood sample was provided as a source of DNA for srWGS. AoU performed the srWGS analysis of the DNA sample. A description of the collection, sequencing process, and genetic data analysis were previously presented [16]. The genes analyzed in this study were based on previous findings in understanding critical steps in the KP [8,9,11,13,14,15]. Eighteen SNPs were selected based on their known association with human diseases to assess the four genes and determine if a higher variant frequency was seen in SUD (Table 2) [11]. Boros et al. presented a review of the SNPs of the kynurenine pathway that had previously been identified with multiple disease states [11]. The AoU database was searched, and 18 SNPs that were already characterized in the database were selected. The polymorphisms of the *TDO2*, *IDO1/2*, and *KMO* genes were evaluated to determine if there was a higher frequency of variants in patients diagnosed with SUD compared to a control cohort. The frequency of participants with at least one copy of a variant in the 18 regions listed in Table 3 was calculated using the cohort builder, genomics, and SNP/Indel filters.

### 2.4. Statistical Analysis

To summarize the demographic information, total or mean ± standard deviation (SD) was used to present the descriptive information of the population (Table 4). For association analysis, the Chi-square (χ^2^) test was used to compare the frequency of variants of each polymorphism in cases and controls. The correlation was evaluated between each cohort. An uncorrected *p*-value cutoff of 0.05 was considered significant for the association test. Statistical analysis was completed using GraphPad Prism (Ver. 10.3.0).

## 3. Results

Demographic data of the cohorts are listed in Table 4 by age, self-reported race, and sex at birth. There was a total of *n* = 11,731 in the use cohorts; alcohol use disorder had the largest population (*n* = 4832), then opioid use disorder (*n* = 2759), followed by other use disorder (*n* = 2405), and cannabis use disorder *(n* = 1094), with the smallest being cocaine use disorder (*n* = 641). The control cohort (*n* = 221,389) comprised all AoU cohort participants who had srWGS and were not diagnosed with SUD.

The analysis of the association among *TDO2*, *IDO1/2*, and *KMO* genes with SUD is shown in Table 3. Fourteen of the SNPs evaluated are significantly associated with at least one of the SUD cohorts in the study. Cannabis and cocaine use disorders had the highest number of variants in the polymorphisms evaluated. Alcohol use disorder only had one polymorphism that was significant compared with the control cohort.

Nine polymorphisms of the *TDO2* gene were evaluated for significance in the dataset. Each of the assessed variants for *TDO2* is located in the gene’s promoter region. For two SNPs (rs10857287 and rs1935082), the published minor alleles were more frequent in both the SUD cohorts and the control cohort than the major allele, as seen in previous research [9]. Five of the regions had a significantly different frequency in the cannabis cohort compared to the control. The polymorphisms (rs3755908, rs3775085, and rs3836580) were significantly increased (*p* < 0.0001 for all) in the cannabis cohort, while the rs10857287 and rs3755910 polymorphisms were significantly decreased in the cannabis cohort compared to the control (*p* < 0.0001 and *p* = 0.02, respectively). Frequencies of six of the variants evaluated differed significantly in the cocaine cohort compared to the control; rs3775085 and rs3836580 showed an increase in frequency, while rs3755908, rs10857287, and rs3755910 showed a decrease in frequency compared to the control (*p* < 0.0001 for all five SNPs). Two polymorphisms were significantly higher in the opioid cohort compared to controls (rs3755908 and rs3836580; *p* = 0.008 and 0.0092, respectively). Lastly, the “other” SUD cohort has two polymorphisms significantly higher (rs3775086 and rs3755909; *p* = 0.0386 and 0.0249, respectively), one of which (rs3775086) was not significant in any other cohort was observed. Two of the polymorphisms evaluated demonstrated no significance between the SUD cohorts and the control group in this study (rs17033763 and rs11935082). There was no significant variation between the alcohol cohort and the control for this set of polymorphisms.

Four polymorphisms of the *IDO1* gene and one of the *IDO2* gene were evaluated for significance in the cohorts. The cannabis and cocaine cohorts had the same trend, with rs3509413 and rs9657182 being significantly lower and rs35099072 significantly higher compared to the control cohort. The Chi-square *p*-value for the cannabis cohort for rs35059413, rs9657182, and rs35099072 were 0.0014, 0.0039, and 0.0430, respectively, and for cocaine, all three were <0.0001. The alcohol cohort had a significantly decreased frequency for rs29290115 (*p* = 0.014) compared to the control. One of the SNPs studied demonstrated no significant difference between the cohorts (rs7820268). The opioid and other cohorts had no variants significantly different from the control cohort for the *IDO1* or *IDO2* genes.

Four polymorphisms were evaluated in the *KMO* gene. Again, the cannabis and cocaine cohorts had the same pattern of significantly decreased variant frequencies (rs1053230, rs2275163, and rs1053221) in these cohorts compared to the control (*p* < 0.0001 for all). The opioid cohort also had a significantly decreased frequency for rs2275163 (*p* = 0.0236). The rs1053183 polymorphism was not significantly different in any SUD cohort compared to the control cohort. The alcohol and other cohorts had no variants that varied significantly from the control cohort.

## 4. Discussion

The effects of substance use are increasing from both an economic and medical perspective in the United States. Research on SUD is continually evolving, driven by the shift towards precision medicine. The National Institute of Health (NIH) has started the Helping to End Addiction Long-term Initiative (HEAL), which is funding over 1000 projects nationwide focused on novel therapeutics [17]. To develop novel theories, research has transitioned to understanding the biological and genetic factors contributing to SUD [18]. Limited information on the frequency of KP IDO1/2, TDO2, and KMO genetic variants in SUD cohorts is available. Therefore, this present study is carried out to investigate the association of genetic polymorphisms of *IDO1/2*, *TDO2*, and *KMO* as biomarkers for increased risk of substance abuse in the AoU database. Our results indicated that fourteen of these polymorphisms had a significantly different frequency in at least one of the SUD cohorts than in the control cohort.

The metabolites of the KP play pivotal roles in several processes involved in neurodevelopment and mood regulation roles in biochemical processes. For example, KYNA can help regulate dopamine release and is currently being researched as a potential target for therapeutic intervention in SUD [5,19]. The involvement of TRP metabolism through the KP as it relates to the function of the dopamine reward system supports the search for possible associations between specific genetic variants of enzymes that regulate TRP metabolism as a genetic risk factor of SUD. Multiple enzymes catalyze the KP, and identifying genetic alterations in these enzymes can help to understand further imbalances in the metabolites of the KP and how that can impact recovery from SUD.

TDO2 is the enzyme catalyzing the initial rate-limiting step in TRP metabolism (Figure 1). Variants in the corresponding gene can impact the activity of the encoded protein and subsequently alter the KP metabolites [20]. *TDO2* variants have previously been associated with various disorders, such as Tourette's syndrome, autism, and hypertryptophanemia [11]. In this study, findings for the alcohol cohort were consistent with previous findings from Soichoit et al., which showed that no significant variation in *TDO2* variant frequencies was observed in the alcohol cohort [9]. Alternatively, a significant difference was seen in each of the other SUD cohorts. These SNPs could result in either increased or decreased enzyme activity, leading to altered concentrations of the KP metabolites compared to healthy controls. As an example, it was previously reported by Comings et al. that variants in the *TDO2* promoter region resulted in an increase in KYN in patients with Tourette syndrome, which could also lead to an increase in QA [8]. These variants might also be expected to be increased in SUD cohorts, as SUD has been reported to have higher QA concentrations than individuals without SUD [21]. QA, a neurotoxic metabolite of KP, can induce an increase in the release of dopamine, which can increase the risk of SUD based on the enhanced release of dopamine. The alternate is true with KA, the neuroprotective metabolite, which can decrease dopamine release, helping with SUD recovery [5].

The IDO1 and IDO2 enzymes also catalyze the initial step, converting TRP to KYN in the KP. *IDO1/2* variants have previously been associated with depression, depression treatment outcomes, Crohn’s disease, and systemic sclerosis [11]. Specifically, the rs9657182 SNP has been shown to increase the chances of depression during treatment for infectious disease [22]. Highlighting the role *IDO1/2* variants play in depression and the potential role they could have in SUD. Lee et al. investigated the relationship between Crohn’s disease and *IDO1/2* and found that with the rs35059413 SNP, serum samples had a decrease in KYN concentration and decreased KYN/TRP ratio [21]. Both the cannabis and cocaine cohorts had significantly increased percentages of participants with this polymorphism. This study further identified that variants within the genes encoding the enzymes of the KP can cause imbalances in the metabolites, potentially increasing the individual’s chance of multiple diseases, one of which being SUD. Two of the polymorphisms of the IDO1 gene are missense mutations. These have been identified as having diminished enzyme activity [11]. Neither the opioid nor the other cohorts had significant frequency differences in the polymorphisms of these genes.

KMO catalyzes the reaction of kynurenine through the neurotoxic pathway, resulting in QA and NAD+. A polymorphism in the *KMO* gene, rs1053230, has previously been identified to be associated with bipolar disorder [15]. Lavebratt et al. found that the variant C allele of the rs1053230 polymorphism is associated with lower KMO expression, increasing KA concentration. In this study, the cannabis and cocaine cohorts demonstrated a significantly decreased frequency of the variant, likely resulting in less of the KA metabolite being created and more of the neurotoxic metabolite, QA, forming in these individuals. This finding may further explain the habitual use of addictive substances because of the increase in dopamine release when QA is increased and KA is decreased. Neither the alcohol nor the “other” SUD cohort had a significant difference in the KMO gene. Studies have previously found imbalances in KP metabolites, and an increase in QA, a neurotoxic compound, has been seen in serum samples of individuals with SUD [5,21]. One explanation for the elevated QA is the significant difference in gene variance found in SUD cohorts. This increased knowledge about the genetic variants in the KP more frequently associated with SUD and their resulting impact on the metabolites of the KP pathway can aid in identifying therapeutic interventions designed to reestablish balance in the KP metabolites. Research in SUD has recently been focused on evaluation if targeting the KP for therapeutic intervention in SUD is an option [21]. Determining genetic variants of enzymes in the KP in SUD could lead to personalized pharmacotherapy, where genetic testing could be performed, and treatments are tailored to an individual’s metabolic. Targeted therapies could include modulators of specific enzymes to restore balance in the pathway and reduce neuroinflammation, potentially decreasing relapse and withdrawal symptoms. Additionally, neuroprotective agents or anti-inflammatory treatments could be developed to protect against the neurotoxic effects of metabolites such as quinolinic acid, improving cognitive function and overall treatment outcomes. One example is Ro 61-8048, a KMO inhibitor, which has shown promising results as a therapeutic option to help prevent relapse in cannabis and cocaine use disorder [18,19].

This study used a retrospective cohort from the AoU database, providing a large sample cohort with extensive clinical and genomic data. The strengths include the low cost of the study since the information is readily available, and the genomic frequencies were evaluated using data cohorts, which is lower in cost compared to extracting the data using Python or R. A study weakness is that haplotypes were not assessed within the cohorts and should be considered in follow-up research to understand penetrance better. Additionally, future research could determine if further correlations exist with the age of onset, the pattern of use habit, polysubstance abuse, and the timeframe of use to see if these cohort subsets strongly correlate to the genetic variants. Additionally, environmental factors that impact SUD, such as stress, trauma, peer influence, family environment, and socioeconomic status, were not evaluated in this study and could be a confounding factor. Future research could benefit from a prospective study where screening for potential confounding factors and personal surveys designed specifically for identifying potential environmental factors would eliminate the chance of these variables impacting the results. Further research could also exclude participants who have bipolar disorder, schizophrenia, depression, or other diagnoses that could confound the results of the study. These variables could be a reason why the cohorts had different polymorphisms that were seen at significantly different rates and should be controlled for in future studies. This correlation has been seen in different variants that are prevalent in SUD [19]. This research would further the understanding of this molecular mechanism on SUD.

Another limitation of the study was that KP metabolite concentrations for the participants were unavailable for the dataset. Limited research has been completed to determine the relationship of the genetic variants to the concentration of these metabolites in serum [11,12,14,15,20,21]. Based on these studies, expected changes in these metabolites could be hypothesized based on the presence of different variants but could not be confirmed. Additionally, while there is limited research on the alterations of the kynurenine pathway in these SUD cohorts, further studies can explore how alcohol, cannabis, cocaine, opioids, and other addictive substances influence the kynurenine pathway to gain a more comprehensive understanding of their relationships. Studies combining genetic variation data with observed KP metabolite concentrations could provide a further understanding of the association of these genetic variants. Understanding if these variants significantly differ in the SUD cohorts, alter or disrupt the enzyme regulation and activity, or cause protein instability, will help further understand these variants’ impact. This provides further knowledge on the potential for therapeutic targets and drugs that can alter the pathway to regulate the metabolites.

## 5. Conclusions

In conclusion, this study highlights the association of genetic variants in the KP within SUD cohorts, suggesting a potential molecular mechanism contributing to SUD. Identifying specific polymorphisms associated with altered enzyme function or metabolite levels provide a foundation for understanding how disruptions in this pathway may influence neurobiological processes involved in SUD. These findings suggest potential biomarkers for developing and monitoring future therapeutic interventions. Identifying the genetic alterations underlying certain diseases can enhance diagnosis and treatment in precision medicine. This knowledge can aid in drug discovery and the development of treatment regimens that either replace missing components or restore balance to altered KP metabolites. The findings from this study can potentially drive significant advancements in treating patients with SUD. 

## Figures and Tables

**Table 1:** List of ICD-10 codes used in the cohort build.

ICD-10	Description
F10	Alcohol-Related disorders
F11	Opioid-Related Disorders
F12	Cannabis-related disorders
F13	Sedative, hypnotic, or anxiolytic-related disorders
F14	Cocaine related disorders
F15	Other stimulant-related disorders
F16	Hallucinogen related disorders
F17	Nicotine dependence
F18	Inhalant related disorders
F19	Other psychoactive substance-related disorders

**Table 2:** Description of polymorphisms analyzed.

Polymorphism	Gene	Consequence	Variant Type
rs3755908	*TD02*	upstream gene variant	SNV
rs3775085	*TD02*	upstream gene variant	SNV
rs17033763	*TD02*	upstream gene variant	SNV
rs3836580	*TD02*	upstream gene variant	insertion
rs10857287	*TD02*	upstream gene variant	SNV
rs11935082	*TD02*	upstream gene variant	SNV
rs3775086	*TD02*	upstream gene variant	SNV
rs3755909	*TD02*	upstream gene variant	SNV
rs3755910	*TD02*	upstream gene variant	SNV
rs35059413	*IDO1*	missense variant	SNV
rs9657182	*IDO1*	intron variant	SNV
rs35099072	*IDO1*	missense variant	SNV
rs7820268	*IDO1*	missense variant	SNV
rs2929115	*IDO2*	Unknown	SNV
rs1053230	*KMO*	missense variant	SNV
rs2275163	KMO	intron variant	SNV
rs1053221	KMO	3’ UTR variant	SNV
rs1053183	KMO	3’ UTR variant	SNV

**Table 3:** Association of polymorphisms in the enzymes IDO1/2, TDO, and KMO with substance use disorder in the All of Us cohort.

Polymorphism	Gene	Alcohol Cohort (*n* = 4832)		Cannabis Cohort (*n* = 1094)		Cocaine Cohort (*n* = 641)		Opioid Cohort (*n* = 2759)		“Other” Cohort (*n* = 2405)	
		Freq	*p*-Value	Freq	*p*-Value	Freq	*p*-Value	Freq	*p*-Value	Freq	*p*-Value
rs3755908	TD02	1462 (30.25%)		416 (38.03%)	<0.0001 *	93 (14.51%)	<0.0001 *	904 (32.77%)	0.0082 *	770 (32.02%)	
rs3775085	TD02	1461 (30.23%)		416 (38.03%)	<0.0001 *	293 (45.71%)	<0.0001 *	904 (32.77%)		771 (32.06%)	
rs17033763	TD02	1155 (23.9%)		292 (26.69%)		147 (22.93%)		677 (24.54%)		591 (24.57%)	
rs3836580	TD02	1461 (30.23%)		416 (38.03%)	<0.0001 *	293 (45.71%)	<0.0001 *	903 (32.73%)	0.0092 *	770 (32.02%)	
rs10857287	TD02	4393 (90.91%)		927 (84.74%)	<0.0001 *	474 (73.95%)	<0.0001 *	2462 (89.24%)		2190 (91.06%)	
rs11935082	TD02	4829 (99.93%)		1094 (100%)		641 (100%)		2758 (99.96%)		2402 (99.88%)	
rs3775086	TD02	702 (14.52%)		165 (15.08%)		89 (13.88%)		419 (15.19%)		393 (16.34%)	0.0386 *
rs3755909	TD02	568 (11.75%)		121 (11.06%)		38 (5.93%)	<0.0001 *	337 (12.22%)		331 (13.76%)	0.0249 *
rs3755910	TD02	298 (6.17%)		46 (4.21%)	0.0200 *	11 (1.72%)	<0.0001 *	145 (5.26%)		130 (5.41%)	
rs35059413	IDO1	82 (1.7%)		34 (3.11%)	0.0014 *	41 (6.4%)	<0.0001 *	64 (2.32%)		40 (1.66%)	
rs9657182	IDO1	3987 (82.51%)		940 (85.92%)	0.0039 *	586 (91.42%)	<0.0001 *	2307 (83.63%)		2022 (84.08%)	
rs35099072	IDO1	26 (0.54%)		10 (0.91%)	0.0430 *	11 (1.72%)	<0.0001 *	21 (0.76%)		15 (0.62%)	
rs7820268	IDO1	2323 (48.08%)		529 (48.35%)		290 (45.24%)		1331 (48.24%)		1190 (49.48%)	
rs2929115	IDO2	4729 (97.87%)	0.0014 *	1085 (99.18%)		638 (99.532%)	0.0261 *	2720 (98.59%)		2365 (98.34%)	
rs1053230	KMO	1418 (29.35%)		246 (22.49%)	<0.0001 *	87 (13.57%)	<0.0001 *	770 (27.91%)		662 (27.53%)	
rs2275163	KMO	2362 (48.88%)		439 (40.13%)	<0.0001 *	249 (38.85%)	<0.0001 *	1261 (45.71%)	0.0236 *	1169 (48.61%)	
rs1053221	KMO	1158 (23.97%)		201 (18.37%)	<0.0001 *	81 (12.64%)	<0.0001 *	629 (22.8%)		560 (23.28%)	
rs1053183	KMO	2350 (48.63%)		563 (51.46%)		346 (53.98%)		1351 (48.97%)		1177 (48.94%)	

* Statistical Significance.

**Table 4:** Demographics of study cohort.

Variables	Cohort Breakdown					
	Alcohol Use Disorder	Cannabis Use Disorder	Cocaine Use Disorder	Opioid Use Disorder	“Other” Use Disorder	Control Cohort
	*n* = 4832	*n* = 1094	*n* = 641	*n* = 2759	*n* = 2405	*n* = 221,389
Age (years)	56 ± 14	47 ± 16	59 ± 10	60 ± 13	53 ± 14	56 ± 17
Sex at Birth						
Male	2954	455	362	1163	1114	82,047
Female	1787	607	248	1528	1238	134,863
Unknown	91	32	31	68	53	4479
Self-Reported Race						
Asian	45	5	2	14	16	7467
Black or AA	1014	438	432	733	472	44,096
Middle Eastern or North African	16	6	-	6	8	1321
Native Hawaiian or Other Pacific Islander		-	-	-	11	238
White	2792	367	92	1504	1218	117,861
Unknown	965	278	115	502	680	50,406

## Data Availability

The raw data supporting the conclusions of this article will be made available by the authors upon request.
